# Methanolic *Moringa oleifera* leaf extract protects against epithelial barrier damage and enteric bacterial translocation in intestinal I/R: Possible role of caspase 3

**DOI:** 10.3389/fphar.2022.989023

**Published:** 2022-09-23

**Authors:** O A. Afolabi, T M. Akhigbe, R E. Akhigbe, B A. Alabi, O T. Gbolagun, M E. Taiwo, O O. Fakeye, E O. Yusuf

**Affiliations:** ^1^ Department of Physiology, Ladoke Akintola University of Technology, Ogbomoso, Oyo, Nigeria; ^2^ Department of Agronomy, Osun State University, Osogbo, Osun, Nigeria; ^3^ Reproductive Biology and Toxicology Research Laboratory, Oasis of Grace Hospital, Osogbo, Osun, Nigeria; ^4^ Department of Pharmacology, Bowen University, Ogbomoso, Nigeria

**Keywords:** Apoptosis, bacterial translocation, hepatic function, inflammation, ischaemia/reperfusion, *Moringa oleifera*, oxidative stress, torsion/detorsion

## Abstract

**Background:** Activation of caspase 3 has been implicated in the pathogenesis of I/R injury in various organs, but there is a paucity of data on its role in IIRI. Also, no reports were found on the beneficial role of methanolic *Moringa oleifera* leaf extract (MMOLE) in IIRI. This study investigated the involvement of caspase 3 in IIRI, and the impact of MMOLE in IIRI.

**Methods:** Male Wistar rats were randomized into five groups; the sham-operated group that was sham-operated and received 0.5 ml of distilled water for 7 days prior to sham surgery, and the IIRI, febuxostat (FEB) +IIRI, low dose MMOLE (LDMO)+IIRI, and high dose MMOLE (HDMO)+IIRI groups that underwent I/R and also received 0.5 ml of distilled water, 10 mg/kg of febuxostat, 200 mg/kg of MMOLE, and 400 mg/kg of MMOLE respectively for 7 days prior to I/R. Markers of hepatic function, oxidative stress, and inflammation as well as enteric bacterial translocation and histoarchitecture integrity of intestinal and hepatic tissues were evaluated. The bioactive components of MMOLE were also determined by GC-MS.

**Results:** As revealed by GC-MS, the active bioactive components of MMOLE were thiosemicarbazone, hydrazine, 1,3-dioxolane, octanoic acid, 1,3-benzenediamine, 9-octadecenoic acid, oleic acid, nonadecanoic acid, 3-undecanone, phosphonic acid, and cyclopentanecarboxylic acid. MMOLE alleviated IIRI-induced rise in intestinal and hepatic injury markers, malondialdehyde, TNF-α, IL-6, and myeloperoxidase activities. MMOLE improved IIRI-induced suppression of reduced glutathione, thiol and non-thiol proteins, and superoxide dismutase, catalase and glutathione peroxidase activities. These were associated with suppression of IIRI-induced caspase 3 activity and bacterial translocation. Histopathological evaluation revealed that MMOLE attenuated IIRI-induced alterations in intestinal and hepatic histoarchitecture integrity. MMOLE also militated against increased absolute and relative intestinal and hepatic weight, intestinal and hepatic injuries, epithelial mucosal barrier dysfunction, and enteric bacterial translocation associated with IIRI by downregulating oxidative stress-mediated activation of caspase 3.

**Conclusion:** IIRI is associated with a rise in caspase 3 activity. Also, MMOLE confers protection against IIRI, possibly due to its constituent bioactive molecules, especially hydrazine, 9-octadecenoic acid, 1,3-dioxolane, oleic acid, and nonadecanoic acid.

## 1 Introduction

Intestinal ischaemia/reperfusion injury (IIRI) is a life-threatening condition with high morbidity and mortality. It is associated with several pathologies such as small bowel transplantation, septic shock, cardiopulmonary resuscitation, and mesenteric artery embolization (Wen et al., 2020). IIRI involves a series of pathological events stimulated by abrupt disruption of blood flow and subsequent establishment of blood flow, leading to increased generation of reactive oxygen species (ROS), inflammation, and apoptosis (Liu et al., 2020). This cascade of events alters the integrity of the intestinal mucosal barrier, thus facilitating bacterial translocation (BT) and the release of endotoxins into the liver and eventually into the systemic circulation to affect remote organs such as the lungs and the kidneys (Park et al., 1990; Kubes, 1993). This may eventually culminate in systemic inflammatory response syndrome (SIRS), multiple organ dysfunction (MODS), and multiple organ failure (MOF) (Feng et al., 2017).

Although the mechanisms associated with IIRI and BT is yet to be fully understood, it has been demonstrated that oxidative stress (an imbalance between ROS generation and scavenging) (Akhigbe and Ajayi, 2021) and pro-inflammatory cytokines play a key role (Mallick et al., 2004). Hence, the use of antioxidants with anti-inflammatory activities, such as *M. oleifera*, may be useful in the management of IIRI and its attendant complications.

*M. oleifera,* a member of the family Moringaceae popularly called drumstick, is a small to medium sized tree that is about 10–15 m high and is widely cultivated in Asia, West Indies, South America, and Nigeria (Sreelatha and Padma, 2009). In Nigeria, it is known by different names; *‘Okwe oyibo’* in Igbo, *‘Gawara’ or ‘Habiwal’* in Hausa and *‘Adagba maloye’* or ‘*Ewe Igbale*’ in Yoruba. Studies have shown that various parts of the plant (especially the leaves, seeds and roots) have medicinal value. *Moringa oleifera* has been demonstrated to possess analgesic, antipyretic, anti-diabetic, and hypotensive properties (Saka et al., 2020). It has also been reported to exert antioxidant (Charoensin, 2014; Saka et al., 2020), anti-inflammatory (Cheenpracha et al., 2010), and antimicrobial/antibacterial (Rahman et al., 2009) activities. Although various parts of the plants exert these activities, the leaves have been reported to be commonly used due to its wide range of beneficial biological activities (Xu et al., 2019). The biological activities of *Moringa oleifera* have been attributed to its phytochemical constituents, viz. carotenoids, vitamins, minerals, amino acids, saponins, terpenoid, sterols, glycosides, alkaloids, flavonoids, tannins, anthraquinones, and phenolics (Siddhuraju and Becker, 2003; Anwar et al., 2007; Saka et al., 2020). Although, reports on the effect of *Moringa oleifera* on caspase 3-mediated apoptosis are scarce, it is likely that *Moringa oleifera-*mediated maintenance of redox balance and modulation of cytokines suppresses caspase 3 activity. Thus, we speculated that *Moringa oleifera* prevents IIRI-induced hepato-intestinal injury and BT by inhibiting oxidative stress, inflammation, and apoptosis.

Therefore, this study evaluated the effect of methanolic *Moringa oleifera* leaf extract (MMOLE) in IIRI and IIRI-induced BT, and also confirmed whether or not suppression of oxidative stress, inflammation, and caspase 3-mediated apoptosis mediate the effect of MMOLE in IIRI.

## 2 Materials and methods

### 2.1 Plant collection

Fresh leaves of *M. oleifera* were harvested at California area, Ogbomoso, Oyo State, Nigeria (8.1333°N, 4.2356°E). Plants were collected monthly throughout year 2020 and mixed together to avoid the influence of seasonal variation. The plant was identified and authenticated by Dr. Mrs. Ogundola of the Botany Unit, Department of Pure and Applied Biology, Ladoke Akintola University of Technology, Ogbomoso. The name of the botanical was confirmed on http://www.theplantlist.org (accessed on 20 July 2021). A voucher specimen, LHO 616, was obtained and kept in the Herbarium at the Department of Botany, Ladoke Akintola University of Technology, Ogbomoso, Nigeria.

### 2.2 Preparation of plant extract

Methanolic *M. oleifera* leaf extract (MMOLE) was made as earlier reported (Sofowora, 1993; Ogundola et al., 2021). Fresh leaves of *Moringa oleifera* were air-dried for 2 weeks and pulverized using electric blender. About 500 g of the obtained sample was soaked in 70% methanol for 72 h (3 days). After 3 days, the samples were filtered with muslin paper to separate the residue from the filtrate. The filtrate was then poured inside the round bottom flask of the Soxhlet apparatus and heated for 1 hour at 60°C, then poured inside a beaker and placed in a water bath for concentration at 100°C for 24 h. The obtained yield was 11.72%.

### 2.3 Gas chromatography-mass spectrophotometricanalysis

The constituent bioactive molecules of methanolic *M. oleifera* leaf extract were identified by gas chromatography-mass spectrophotometric (GC-MS) analysis following established methods (Mamza et al., 2012). The database of National Institute of Standard and Technology (NIST) was employed in interpreting the mass spectrum of GC-MS to ascertain the name, molecular weight and structure of the obtained bioactive molecules.

### 2.4 Experimental animals

The present study was conducted in the animal house of the Department of Physiology, Ladoke Akintola University of Technology, Ogbomoso, Nigeria. Ethical approval was obtained from the Ethics Review Committee of the Faculty of Basic Medical Sciences of the institution. Fifty adult male Wistar rats of similar weight (190 ± 5 g) were used for this study. Animals were allowed to feed on standard rat chow and drink water ad’libitum. Animals were humanely cared for in accordance with the guidelines for the care and use of laboratory animals as published by the US National Institutes of Health (NIH Publication No. 85-23, revised 1996).

### 2.5 Experimental design

Animals were allowed to acclimatize for 2 weeks, and then randomized into five groups (*n* = 10). The sham group was sham-operated and received 0.5 ml of distilled water for 7 days prior to the sham operation, while the IIRI, febuxostat (FEB) +IIRI, low dose methanolic *Moringa oleifera* leaf extract (LDMO)+IIRI, and high dose methanolic *Moringa oleifera* leaf extract (HDMO)+IIRI groups underwent I/R procedure. In addition to the I/R procedure, IIRI, FEB + IIRI, LDMO + IIRI, and HDMO + IIRI received 0.5 ml of distilled water, 10 mg/kg of febuxostat, 200 mg/kg of methanolic *M. oleifera* leaf extract, and 400 mg/kg of methanolic *M. oleifera* leaf extract respectively for 7 days prior to I/R procedure. Febuxostat was used as a standard control drug with anti-inflammatory and antioxidant properties that protect against I/R injury (Tsuda et al., 2012; Saban-Ruiz et al., 2013). The drugs were administered *via* gavage. The doses of febuxostat (Khames et al., 2017) and *M. oleifera* (Kirisattayakul et al., 2013) were as previously reported.

IIRI was induced as previously reported (Yildiz et al., 2009) with some modifications. Animals were weighed and anesthetized with 10 mg/kg of Xylazine and 50 mg/kg of Ketamine. The abdomen was cleaned with 10% povidone iodine, a ventral midline incision was made on the abdomen and the intestine was mobilised. About Five 5 cm of the ileum was measured proximally from the illio-cecal junction and a further 5 cm measured proximal to the first 5 cm. The most proximal 5 cm of ileum was folded on itself and twisted 720^0^clockwise. The twisted ileum was then anchored to the anterior abdominal wall by placing a 3-0 chromic suture round it and through the avascular part of the mesentery. The abdomen was then closed lightly with 3-0 chromic suture after which the animal was left for 45 minutes. At the expiration of the 45 min of ischemia, the abdomen was reopened. Loss of pulsation was observed to confirm that ischemia has occurred. Reperfusion was induced by reopening the abdomen, untwisting the ileum and closing the abdomen again. The animals were then left for 24 h.

### 2.6 Sacrifice and tissue collection

After 24 h of reperfusion, animals were euthanized, blood was obtained via retro-orbital vein, and the reperfused intestine and liver were immediately harvested, and weighed after separating adhering structures. The relative intestinal weight was determined as the weight of the intestine divided by the body weight multiplied by 100, while the relative hepatic weight was determined as the weight of the liver divided by the body weight multiplied by 100.

Portions of the intestinal and hepatic tissues were homogenized in appropriate volume of cold phosphate buffer saline using a glass homogenizer. The homogenates were centrifuged at 10,000 g for15 min in cold centrifuge at 4°C to obtain the supernatant fractions.

Also, portions of the intestinal and hepatic tissues were obtained and fixed in 10% formalin-phosphate-buffered saline at 4°C overnight for histopathological examination.

### 2.7 Biochemical analyses

#### 2.7.1 Haematological indices

A drop of blood was used to prepare a peripheral blood smear. The peripheral blood smear was stained with modified Wright Geimsa using an automated slide stainer (Hematek, Miles, Elkhart, IN). The peripheral blood smear was used to determine red blood cell count (RBC), platelet count, and white blood cell count (WBC) and differentials. The remaining blood sample was subjected to complete/full blood count analysis with an automated hematology instrument (Abbott Cell-Dyn 3500 Hematology Analyzer, Abbott Labs, Abbott Park, IL) for haematocrit ount (Hb), packed cell volume (PCV), mean corpuscular volume (MCV), mean corpuscular haemoglobin concentration, (MCHC), platelet count and differential white blood cell counts for lymphocytes, and granulocytes.

#### 2.7.2 Tissue injury markers

The activities of aspartate transaminase (AST), alanine transferase (ALT), and gamma-glutamyl transferase (GGT) in the intestinal and hepatic tissue were assayed by spectrophotometry as previously reported (Saka et al., 2011).

#### 2.7.3 Markers of oxidative stress and antioxidant levels

The intestinal and hepatic concentrations of malondialdehyde (MDA) and reduced glutathione (GSH) (Akhigbe et al., 2021), and the activities of superoxide dismutase (SOD), catalase, and glutathione peroxidase (GPx) (Hamed et al., 2021; Hamed et al., 2022) in the intestinal and hepatic tissues were assayed by spectrophotometry as previously reported. Thiol and non-thiol proteins were assayed by colorimetry as earlier reported (Jocelyn, 1987; Hu, 1994).

#### 2.7.4 Markers of inflammation

Myeloperoxidase (MPO) activities in the intestinal and hepatic tissues were determined by colorimetric methods as earlier documented (Hamed et al., 2021). The intestinal and hepatic levels of tumour necrotic factor-α (TNF-α) and interleukin-6 (IL-6) were determined using ELISA kits (Elabscience Biotechnology Inc., United States) according to the manufacturers’ guidelines.

#### 2.7.5 Marker of apoptosis

The activities of caspase 3 in the intestinal and hepatic tissues were assayed using ELISA kits (Elabscience Biotechnology Inc., United States) according to the manufacturers’ guidelines.

### 2.8 Microbiological analysis

Bacterial translocation was determined as previously reported (Ozkan et al., 2009) under strict sterile conditions. Briefly, some portions of the intestinal and hepatic tissues were cut into pieces with a sterile blade and added to 1 ml of Mueller-Hinton Broth. The samples were homogenized and about 100 µl of each sample was inoculated into nutrient agar (NA), eosin-methylene blue (EMB), and Mac Conkey agar (MCCA) for colony counts. The cultures were incubated for 48 h and observed for the presence of growth under either aerobic or anaerobic conditions.

### 2.9 Histopathological analysis

Formalin-phosphate-buffered saline-fixed intestinal and hepatic tissues were dehydrated and embedded in paraffin wax. About 5 µm thick sections were cut and stained with hematoxylin-eosin and examined under light microscope by two pathologists who were blinded to the study protocol. Photomicrographs were taken at ×100 and ×400 magnifications.

Intestinal (Chiu et al., 1970) and hepatic (Eckhoff et al., 2002) histomorphological damage was scored using the Chiu and Eckhoff’s score respectively. The mean values for each variable obtained by both pathologists were used as the values of the variables.

The digital photomicrographs obtained were imported unto ImageJ Software (NIH, Bethesda, MD, United States) with specific plugins for quantification. The villus height and crypt depth were determined by two experts. The mean values for each variable obtained by both experts were used as the values of the variables.

### 2.10 Statistical analysis

GraphPad Prism (Versions 7.00) was used for data analysis. D’Agostino Pearson Omnibus and Shapiro–Wilk normality tests were conducted to ascertain that the data set were normally distributed. Comparisons of mean values were made by Analysis of variance (ANOVA) followed by Tukey’s posthoc test for pair-wise comparison. Data are presented as means ± standard deviations. A *p* value <0.05 was considered significant.

## 3 Results

### 3.1 Bioactive compounds of methanolic *M. oleifera* leaf extract

Supplementary Table S1 and Supplementary Figure S1 show the bioactive compounds of methanolic *M. oleifera* leaf extract as revealed by GC-MS. It was observed that cyclopentanecarboxylic acid had the highest retention time (18.676 min), followed by phosphonic acid (17.072 min) and 3-undecanone (16.023 min), while thiosemicarbazone had the least (2.631 min) followed by hydrazine (3.987 min) and 1,3-dioxolane (5.379 min). Hydrazine was found to be the highest compound (14.50%), followed by 9-octadecenoic acid (8.74%), 1,3-dioxolane (2.92%), and oleic acid (2.14%). The chemical properties of these organic constituents are shown in Table 1 and the chemical structures are provided in Supplementary Figure S2.

**TABLE 1:** Effect of intestinal ischaemia/reperfusion (I/R) and methanolic *Moringa oleifera* leaf extract on full blood count.

	Sham	IIRI	FEB + IIRI	LDMO + IIRI	HDMO + IIRI
RBC (x 10^12^/L)	6.66 ± 0.29	7.44 ± 0.64^a^	7.52 ± 0.64^a^	8.34 ± 0.20^a,b,c^	7.47 ± 0.12^a,d^
Hb (g/dl)	13.02 ± 0.68	14.78 ± 0.41^a^	14.22 ± 0.48^a^	16.08 ± 0.52^a,b,c^	14.84 ± 0.29^a,d^
PCV (%)	36.4 ± 1.67	39.90 ± 0.68^a^	38.74 ± 1.36^a^	41.24 ± 0.60^a,c^	42.48 ± 0.77^a,b,c^
MCV (fL)	54.69 ± 1.16	53.73 ± 0.65	54.30 ± 1.52	57.50 ± 0.86^a,b,c^	54.24 ± 0.89^d^
MCHC (g/L)	345.20 ± 12.74	354.80 ± 3.39	362.80 ± 7.43^a^	359.60 ± 6.60^a^	366.20 ± 3.74^a^
WBC (10^9^/L)	9.87 ± 0.30	13.03 ± 1.14^a^	5.12 ± 0.56^a,b^	8.28 ± 0.74^a,b,c^	8.24 ± 0.79^a,b,c^
Lymphocytes (%)	74.74 ± 1.45	61.90 ± 1.74^a^	60.30 ± 2.18^a^	63.90 ± 0.69^a,c^	63.08 ± 2.56^a^
Granulocytes (%)	16.83 ± 0.65	27.83 ± 1.91^a^	13.00 ± 1.14^a,b^	22.35 ± 0.71^a,b,c^	26.13 ± 2.09^a,c,d^
Platelets (10^9^/L)	361.00 ± 42.45	335.30 ± 15.95	483.60 ± 33.50^a,b^	469.50 ± 50.33^a,b^	579.80 ± 8.26^a,b,c,d^

^a^ *p* < 0.05 versus sham, ^b^ *p* < 0.05 versus IIRI, ^c^ *p* < 0.05 versus FEB+IIRI, ^d^ *p* < 0.05 versus LDMO+IIRI

Data were analyzed by one-way ANOVA followed by Tukey’s post hoc test. Values are expressed as mean ± SD of 10 rats per group.

### 3.2 Organ weight

**FIGURE 1:**
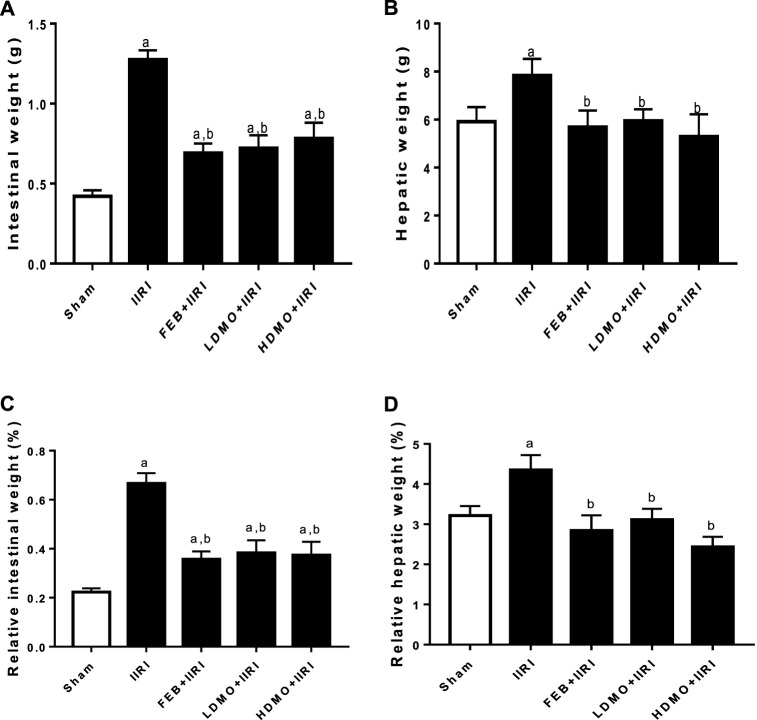
Effect of intestinal ischaemia/reperfusion (I/R) and methanolic *Moringa oleifera* leaf extract on intestinal weight **(A)**, hepatic weight **(B)**, relative intestinal weight **(C)**, and relative hepatic weight **(D)**. IIRI: Intestinal ischaemia/reperfusion injury, FEB: febuxostat, LDMO: low dose *Moringa oleifera,* HDMO: high dose *Moringa oleifera,* ^a^ *p* < 0.05 versus sham, ^b^ *p* < 0.05 versus IIRI. Data were analyzed by one-way ANOVA followed by Tukey’s post hoc test. Values are expressed as mean ± SD of 10 rats per group.

As shown in Figure 1, I/R led to a significant increase in the absolute and relative intestinal and hepatic weight compared with the animals in the sham group. I/R-induced increase in the absolute and relative intestinal and hepatic weight were significantly attenuated by FEB, LDMO, and HDMO. The effects of MMOLE on absolute and relative intestinal and hepatic weight were not dose-dependent.

### 3.3 Intestinal and hepatic injury markers

**FIGURE 2:**
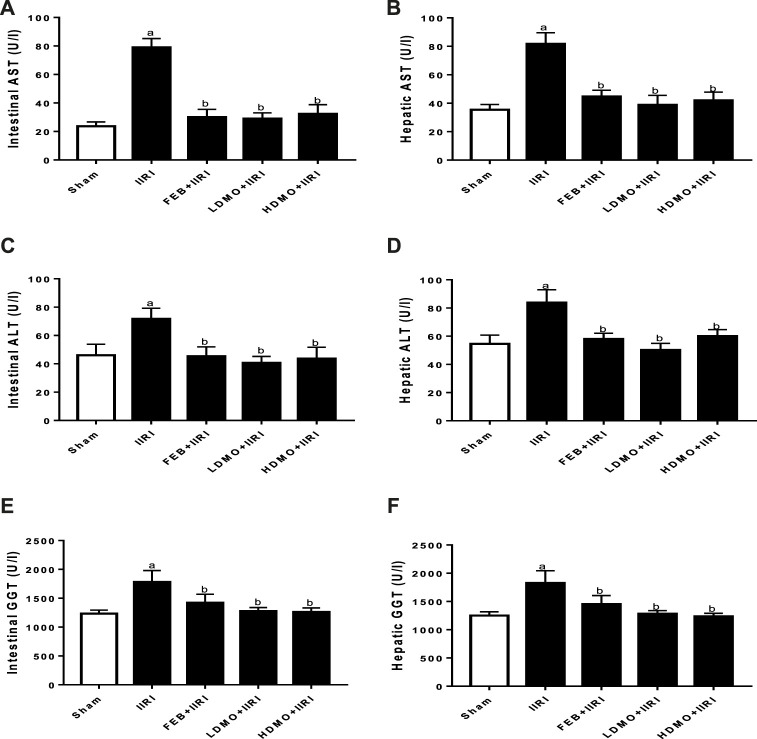
Effect of intestinal ischaemia/reperfusion (I/R) and methanolic *Moringa oleifera* leaf extract on intestinal **(A)** and hepatic aspartate transaminase, AST **(B)**, intestinal **(C)** and hepatic alanine transaminase, ALT **(D)**, and intestinal **(E)** and hepatic gamma-glutamyltransferase, GGT **(F)**. IIRI: Intestinal ischaemia/reperfusion injury, FEB: febuxostat, LDMO: low dose *Moringa oleifera,* HDMO: high dose *Moringa oleifera,* ^a^ *p* < 0.05 versus sham, ^b^ *p* < 0.05 versus IIRI. Data were analyzed by one-way ANOVA followed by Tukey’s post hoc test. Values are expressed as mean ± SD of 10 rats per group.

To investigate the effect of IIRI and MMOLE on tissue injury markers, AST, ALP, and GGT activities were estimated (Figure 2). It was noted that intestinal I/R significantly elevated intestinal and hepatic AST, ALP, and GGT activities compared with the sham group. I/R-induced rise in injury markers was significantly attenuated by FEB, LDMO, and HDMO. The effects of MMOLE on these injury markers were not dose-dependent.

### 3.4 Markers of oxidative stress and inflammation

**FIGURE 3:**
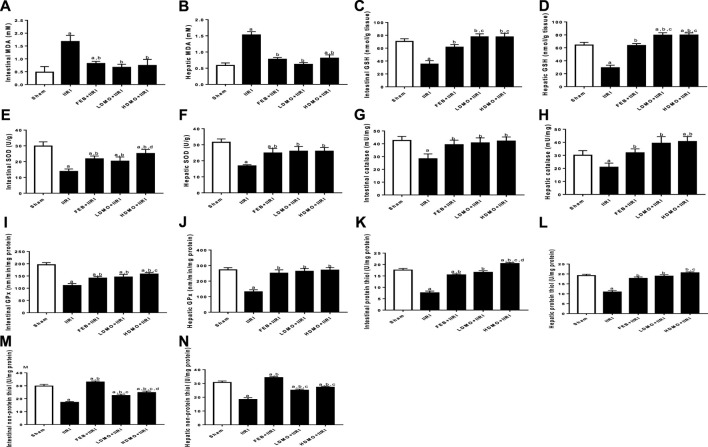
Effect of intestinal ischaemia/reperfusion (I/R) and methanolic *Moringa oleifera* leaf extract on markers of oxidative stress in the intestinal and hepatic tissues. MDA: Malondialdehyde, GSH: reduced glutathione, SOD: superoxide dismutase, GPx: glutathione peroxidase. IIRI: Intestinal ischaemia/reperfusion injury, FEB: febuxostat, LDMO: low dose *Moringa oleifera,* HDMO: high dose *Moringa oleifera,* ^a^ *p* < 0.05 versus sham, ^b^ *p* < 0.05 versus IIRI, ^c^ *p* < 0.05 versus FEB + IIRI, ^d^ *p* < 0.05 versus LDMO + IIRI. Data were analyzed by one-way ANOVA followed by Tukey’s post hoc test. Values are expressed as mean ± SD of 10 rats per group.

IIRI significantly increased intestinal and hepatic MDA. The observed I/R-led rise in MDA was significantly abrogated by FEB, LDMO, and HDMO. In addition, I/R caused a considerable reduction in intestinal and hepatic GSH, thiol and non-thiol protein concentrations as well as SOD, catalase, and GPx activities. I/R-driven decline in these antioxidants was significantly blocked by FEB, LDMO, and HDMO. Although the effects of MMOLE on enzymatic and non-enzymatic antioxidants were observed to be dose-independent, MMOLE exerted a dose-dependent effect on intestinal levels of thiol and non-thiol protein, and SOD activity (Figure 3).

**FIGURE 4:**
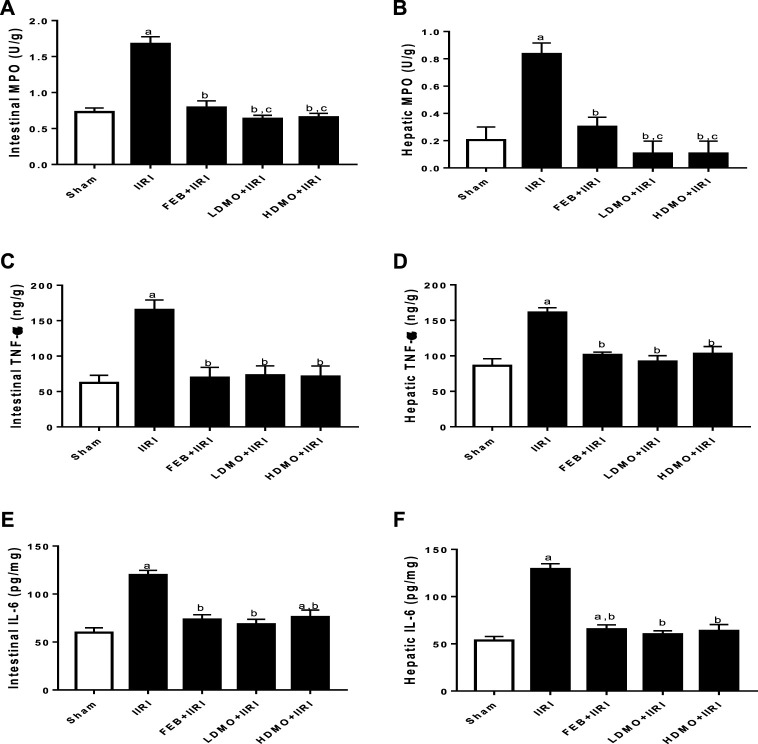
Effect of intestinal ischaemia/reperfusion (I/R) and methanolic *Moringa oleifera* leaf extract on markers of inflammation in the intestinal and hepatic tissues. MPO: Myeloperoxidase, TNF-α: tumour necrotic factor- α, IL-6: interleukin-6. IIRI: Intestinal ischaemia/reperfusion injury, FEB: febuxostat, LDMO: low dose *Moringa oleifera,* HDMO: high dose *Moringa oleifera,* ^a^ *p* < 0.05 versus sham, ^b^ *p* < 0.05 versus IIRI, ^c^ *p* < 0.05 versus FEB + IIRI. Data were analyzed by one-way ANOVA followed by Tukey’s post hoc test. Values are expressed as mean ± SD of 10 rats per group.

In addition, intestinal I/R caused a marked increase in intestinal and hepatic MPO activities and TNF-α and IL-6 concentrations compared with the sham-operated group. I/R-driven rise in these inflammatory markers were significantly ameliorated by FEB, LDMO, and HDMO treatments. The effects of MMOLE on MPO, TNF-α, and IL-6 were dose-independent (Figure 4).

### 3.5 Apoptotic markers

**FIGURE 5:**
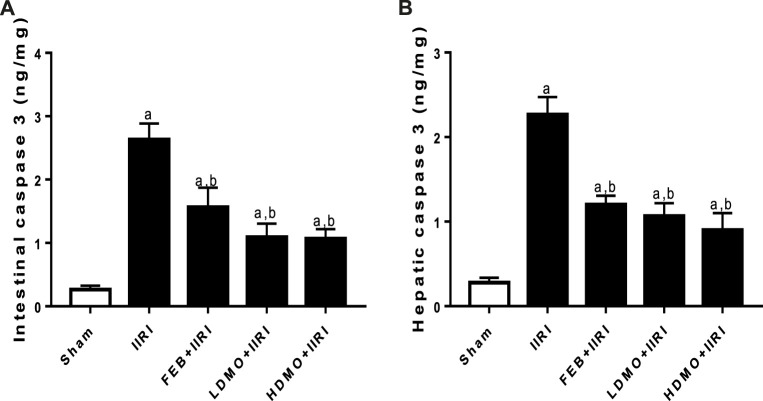
Effect of intestinal ischaemia/reperfusion (I/R) and methanolic *Moringa oleifera* leaf extract on caspase 3 activities in the intestinal and hepatic tissues. IIRI: Intestinal ischaemia/reperfusion injury, FEB: febuxostat, LDMO: low dose *Moringa oleifera,* HDMO: high dose *Moringa oleifera,* ^a^ *p* < 0.05 versus sham, ^b^ *p* < 0.05 versus IIRI, ^c^ *p* < 0.05 versus FEB + IIRI. Data were analyzed by one-way ANOVA followed by Tukey’s post hoc test. Values are expressed as mean ± SD of 10 rats per group.

Intestinal I/R significantly upregulated intestinal and hepatic caspase 3 activities compared with the sham group. I/R-driven rise in caspase 3 activities was significantly abrogated by FEB, LDMO, and HDMO treatments. The effect of MMOLE treatment on caspase 3 activity was not dose-dependent (Figure 5).

### 3.6 Intestinal and hepatic histoarchitecture

**FIGURE 6:**
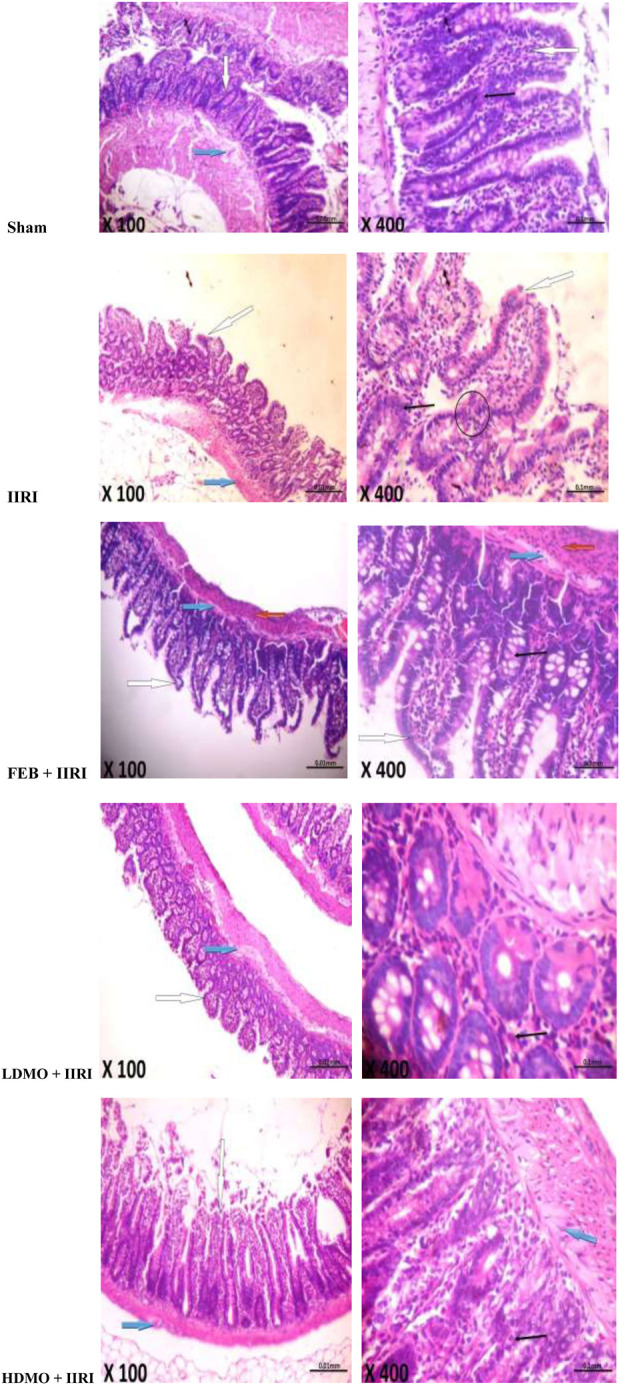
Effect of intestinal ischaemia/reperfusion (I/R) and methanolic *Moringa oleifera* leaf extract on intestinal histoarchitecture. The sham-operated rats showed normal villi from mucosal layer (white arrow). The lumen showed mild lymphocytes infiltration and the propria showed moderate inter-glandular infiltration of inflammatory cells (slender arrow). The submucosal layer was moderarely infiltrated by inflammatory cells (blue arrow). The intestinal ischaemia/reperfusion injury (IIRI) group showed moderately inflamed villi from mucosal layer which is moderately infiltrated by inflammatory cells (white arrow) and neutrophils (circle). The lumen showed severe infiltration of lymphocytes and polymorphs, and the propria showed severe inter-glandular infiltration of inflammatory cells (slender arrow). The submucosal layer appeared moderately to severely infiltrated by inflammatory cells (blue arrow). The febuxostat (FEB) + IIRI showed well preserved villi from mucosal layer (white arrow). The lumen showed mild linflammatory cells infiltration, and the lamina propria showed mild infiltration of inflammatory cells (slender arrow). The submucosal layer appeared normal (blue arrow) and the circular muscle appeared normal (red arrow). The low dose *Moringa oleifera* (LDMO) + IIRI animals showed normal villi from mucosal layer (white arrow). The lumen showed moderate lymphocytes infiltration and the propria showed mild inter-glandular infiltration of inflammatory cells (slender arrow). The submucosal layer appeared normal (blue arrow). The high dose *Moringa oleifera* (HDMO) + IIRI animals showed normal villi from mucosal layer (white arrow). The lumen showed moderate lymphocytes infiltration and the propria showed moderate inter-glandular infiltration of inflammatory cells (slender arrow). The submucosal layer appeared mildly infiltrated (blue arrow).

Histopathological examinations revealed that the sham-operated rats had normal villi from mucosal layer with mild lymphocyte infiltration in the lumen, moderate inter-glandular infiltration of inflammatory cells in the propria showed, and moderate infiltration of inflammatory cells in the submucosal layer. However, IIRI led to moderately inflamed villi from mucosal layer with moderate infiltration by inflammatory cells, severe infiltration of the lumen by lymphocytes and polymorphs, severe inter-glandular infiltration of inflammatory cells in the propria, and moderate to severe infiltration of the submucosal layer by inflammatory cells. The febuxostat FEB + IIRI showed well preserved villi from mucosal layer, mild inflammatory cells infiltration of the lumen and lamina propria, and a well-preserved submucosal layer and circular muscle. MMOLE treatments, in low and high doses, preserved the villi, although there was moderate lymphocytic infiltration and mild inter-glandular infiltration of inflammatory cells of the lumen and propria respectively. Animals treated with LDMO had normal submucosal layer, while those treated with HDMO had mildly infiltrated submucosal layer (Figure 6).

**FIGURE 7:**
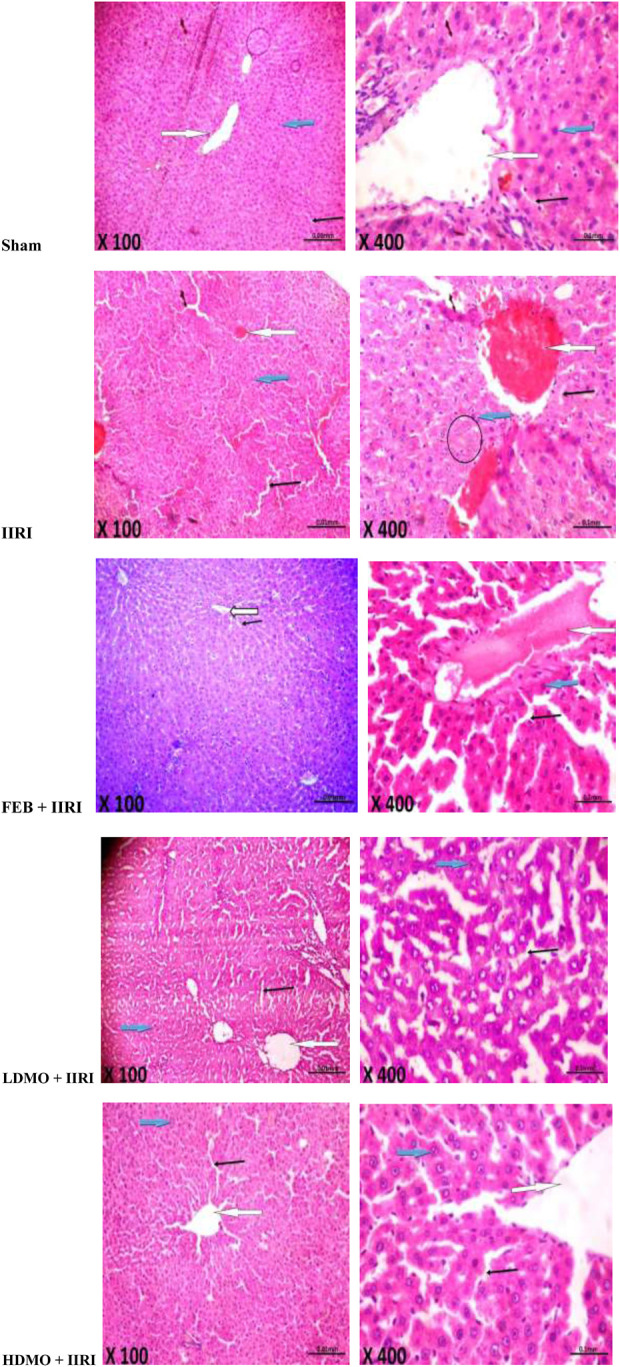
Effect of intestinal ischaemia/reperfusion (I/R) and methanolic *Moringa oleifera* leaf extract on hepatic histoarchitecture. The sham-operated rats showed preserved hepatic histoarchitecture with normal central venules (white arrow). The portal triads and hepatocytes appeared normal (blue arrow), and the sinusoids also appeared normal (slender arrow). The intestinal ischaemia/reperfusion injury (IIRI) group showed moderately congested central venules (white arrow) with normal hepatocytes (blue arrow), mildly dilated sinusoids (slender arrow), and neutrophil infiltration (circle). The febuxostat (FEB) + IIRI rats showed mildly congested central venules (black arrow). However, the hepatocytes appeared normal (white arrow) and the sinusoids appeared mildly dilated (slender arrow). The low dose *Moringa oleifera* (LDMO) + IIRI animals showed normal central venules without congestion (white arrow). The hepatocytes appeared normal (blue arrow) and the sinusoids appeared mildly dilatated (slender arrow). The high dose *Moringa oleifera* (HDMO) + IIRI animals showed normal central venules (white arrow). The hepatocytes appeared normal (blue arrow) and the sinusoids appeared mildly dilated (slender arrow).

In addition, the sham-operated rats showed preserved hepatic histoarchitecture with normal central venules, portal triads, hepatocytes, and sinusoids. IIRI led to moderately congested central venules and mildly dilated sinusoids with normal hepatocytes. FEB + IIRI rats showed mildly congested central venules, mildly dilated sinusoids, with normal hepatocytes. MMOLE treatments showed normal central venules without congestion and normal hepatocytes with mildly dilated sinusoids (Figure 7).

**FIGURE 8:**
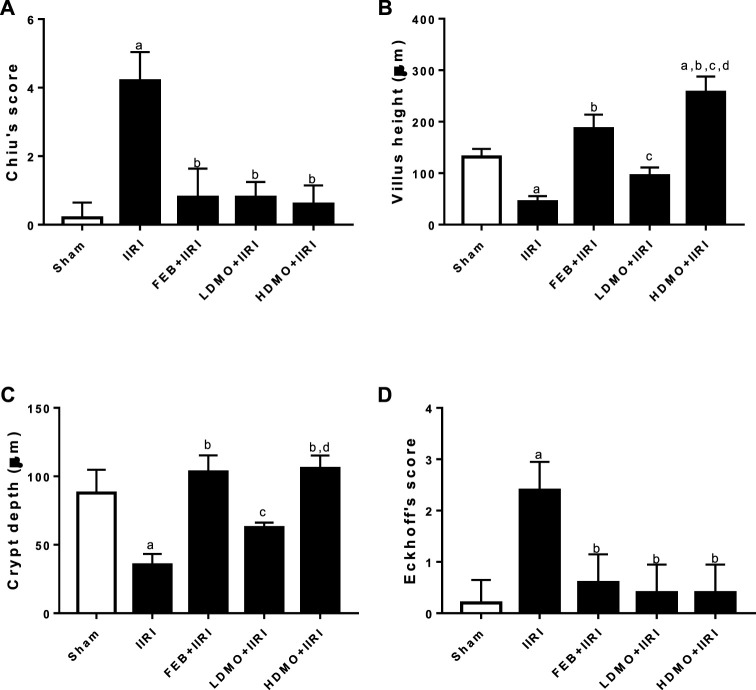
Effect of intestinal ischaemia/reperfusion (I/R) and methanolic *Moringa oleifera* leaf extract on intestinal injury using Chiu’s score **(A)**, villi height **(B)**, crypt depth **(C)**, and hepatic injury using Eckhoff’s score **(D)**. IIRI: Intestinal ischaemia/reperfusion injury, FEB: febuxostat, LDMO: low dose *Moringa oleifera,* HDMO: high dose *Moringa oleifera,* ^a^ *p* < 0.05 versus sham, ^b^ *p* < 0.05 versus IIRI, ^c^ *p* < 0.05 versus FEB + IIRI, ^d^ *p* < 0.05 versus LDMO + IIRI. Data were analyzed by one-way ANOVA followed by Tukey’s post hoc test. Values are expressed as mean ± SD of 10 rats per group.

Figure 8 shows the histomorphological changes in the intestinal and hepatic tissues using the Chiu and Eckhoff’s scores respectively. When compared with the sham-operated, IIRI led to increased intestinal injury (evidenced by increased Chiu’s score) and hepatic injury (evidenced by increased Eckhoff’s score). Administration of FEB, LDMO, and HDMO significantly blunted IIRI-induced intestinal and hepatic injury. The impact of *M. oleifera* on IIRI-driven intestinal and hepatic injury was not dose-dependent. Furthermore, significantly shorter villus length and reduced crypt depth were seen in IIRI rats compared to the sham-operated. IIRI-led reductions in villus length and crypt depth were blunted by FEB and MMOLE administrations. MMOLE improved the villus length and crypt depth in a dose-dependent manner.

### 3.7 Bacterial translocation

**FIGURE 9:**
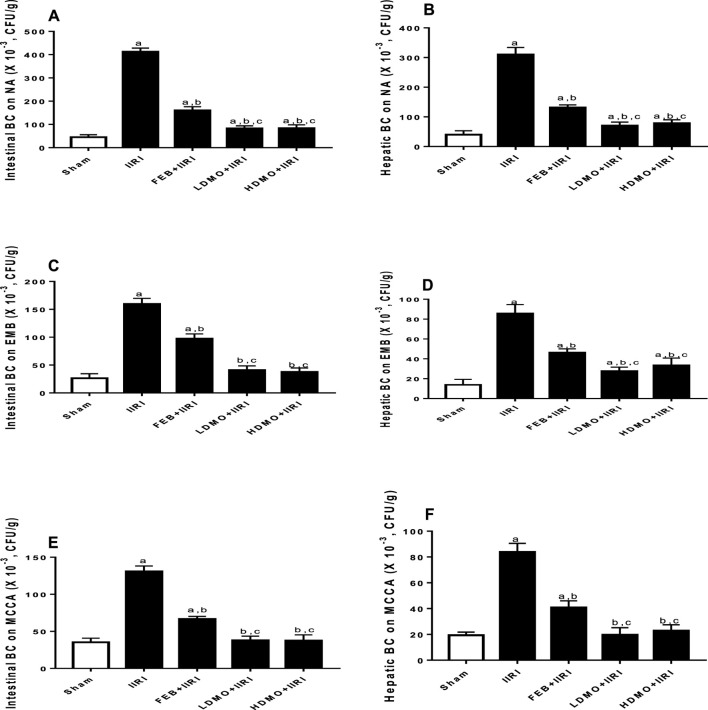
Effect of intestinal ischaemia/reperfusion (I/R) and methanolic *Moringa oleifera* leaf extract on intestinal and hepatic bacterial count (BC) using nutrient agar (NA), eosin-methylene blue (EMB), and Mac Conkey agar (MCCA) culture media for colony count. IIRI: Intestinal ischaemia/reperfusion injury, FEB: febuxostat, LDMO: low dose *Moringa oleifera,* HDMO: high dose *Moringa oleifera,* ^a^ *p* < 0.05 versus sham, ^b^ *p* < 0.05 versus IIRI, ^c^ *p* < 0.05 versus FEB + IIRI. Data were analyzed by one-way ANOVA followed by Tukey’s post hoc test. Values are expressed as mean ± SD of 10 rats per group.

IIRI significantly increased the bacteria count in intestinal and hepatic tissues compared with the sham-operated group in all the culture media used. FEB and MMOLE treatments significantly reduced IIRI-led rise in bacteria count. Interestingly, although the bactericidal activity of MMOLE was not dose-dependent, it significantly reduced bacteria count at the low and high doses when compared FEB treatment (Figure 9).

#### 3.7.1 9 Haematological indices

When compared with the control, IIRI significantly reduced lymphocyte and platelet counts, increased RBC, Hb, PCV, WBC, and granulocytes, but did not alter MCV and MCHC. FEB treatment prevented IIRI-induced rise in WBC and platelet counts and granulocyte. Interestingly, LDMO, but not HDMO, increased RBC count, Hb, and MCHC when compared with the control and IIRI groups; while HDMO and LDMO reduced IIRI-induced rise in WBC count and increased IIRI-induced reduction in platelet count. The effects of MMOLE on RBC, Hb, granulocytes, and platelets were dose-dependent (Table 1).

## 4 Discussion

Caspase 3-mediated apoptosis has been incriminated in the pathogenesis of I/R injury in various organs (Afolabi et al., 2021; Ba et al., 2021; Zhu et al., 2021; Afolabi et al., 2022; Huang et al., 2022; Zhu et al., 2022). However, the role of caspase 3 in the intestinal tract, a bacteria-filled organ, has not been well explored. Also, the possible medicinal benefit of *M. oleifera,* a herbal nutraceutical used in folklore medicine for its anti-inflammatory properties, in IIRI has not been reported. This study revealed that MMOLE-mediated caspase 3 suppression may play a role in its protection against IIRI-induced epithelial barrier dysfunction, bacterial translocation, and hepatic injury. We demonstrated that MMOLE attenuated intestinal mucosal injury, and intestinal and extraintestinal bacterial counts normally seen in IIRI. Downregulation of caspase 3 by MMOLE likely inhibits enteric bacterial intrusion *via* multiple pathways, including enhancement of epithelial barrier integrity via maintenance of cellular antioxidants and direct antimicrobial activity.

Our findings of increased number of bacterial colonies in the liver are in line with previous studies which revealed increased epithelial permeability and bacterial translocation in IIRI (Hsiao et al., 2009; Huang et al., 2010). The increased permeability could have resulted from the disruption of the intestinal barrier arising from oxidative damage to the membranes and subsequent apoptotic denudation of the intestinal epithelium (Hu et al., 2019). The current study showed that MMOLE pretreatment significantly reduced intestinal and hepatic bacterial counts, which was coupled with reduced intestinal mucosal injury and hepatic damage, as well as improved villus length and crypt depth. The attenuation of IIRI-induced bacterial translocation and reduction of WBC and granulocytes (indicators of bacterial infection) by MMOLE may be ascribed to its antimicrobial and/or antioxidant activities. This finding corroborates previous reports on the antimicrobial effects (Rahman et al., 2009) and antioxidant (Charoensin, 2014; Saka et al., 2020) properties of MMOLE. Rahman and his colleagues (2009) reported that the leaf juice and extracts of *M. oleifera* exerted antibacterial activities against tested gram positive and gram negative bacteria, suggestive of the presence of broad spectrum bioactive compounds in the herbal nutraceutical. Abalaka et al. (2012) also demonstrated the activity of *M. oleifera* leaf extract against gram positive and gram negative bacteria, confirming that *M. oleifera* exhibits broad spectrum antibacterial activities. Thus, it is reasonable to infer that MMOLE-mediated reduction in bacterial translocation in IIRI is due to, at least in part, its antibacterial potentials.

The preservation of cellular antioxidants that was associated with preserved intestinal and hepatic cytoarchitecture in MMOLE-treated rats suggests that the botanical conferred cellular protection in IIRI. Cellular antioxidants are integral parts of tissues that protect the cell against ROS-mediated oxidative injury (Hamed et al., 2022). SOD converts superoxide radicals into dioxygen and hydrogen peroxide, which is further broken down into water and molecular oxygen by catalase (Akhigbe et al., 2020). GSH scavenges ROS, while GPx metabolizes hydrogen peroxide and also oxidizes GSH (Aquilano et al., 2014). Sreelatha and Padma (2009) demonstrated that *M. oleifera* leaf extract exhibited strong scavenging effect on 2,2-diphenyl-2-picryl hydrazyl (DPPH) free radical, superoxide radical and nitric oxide radical, thus protects against oxidative damage to biomolecules. Charoensin (2014) also reported the radical scavenging activities of *M. oleifera* leaf extracts. Hence, MMOLE-driven suppression of MDA and upregulation of enzymatic and non-enzymatic antioxidants in IIRI explains, at least partly, the observed preserved intestinal and hepatic cytoarchitecture and maintenance of villus length and crypt depth in MMOLE-treated rats.

Oxidative stress has been established as a cause and/or consequence of inflammation. Oxidative stress triggers the translocation of nuclear factor kappa-light-chain-enhancer of activated B cells (NF-kB) to the nucleus and induces the transcription of several deleterious pro-inflammatory genes (Won et al., 2006; Hamed et al., 2022). In addition, oxidative stress upregulates pro-inflammatory cytokines such as TNF-α and IL-6 that in turn activate NF-kB (Akhigbe and Ajayi, 2020; Hamed et al., 2022), prime neutrophil infiltration (Sheppard et al., 2005), and promote ROS-induced oxidative stress (Akhigbe and Ajayi, 2020). It is therefore plausible to surmise that MMOLE blunted IIRI-induced neutrophil accumulation (evidenced by reduced MPO activity) by suppressing ROS-driven upregulation of TNF-α and IL-6, indicating the anti-inflammatory and antioxidant activities of MMOLE. This agrees with the report of Omodanisi et al. (2017) that demonstrated the anti-inflammatory and antioxidant activities of MMOLE in diabetes-induced nephrotoxic rats. It is also in consonance with the report of Xu et al. (2019) that demonstrated the anti-inflammatory and antioxidant activities of the crude extracts of *M. oleifera* leaves.

In an organ full of commensal bacteria such as the intestine, epithelial barrier disruption by oxido-inflammatory damage is key in extraintestinal bacterial translocation (Kubes, 1993; Liu et al., 2020). It is likely that caspase 3-mediated apoptosis also play a role in altering the epithelial mucosa integrity and promoting microbial dissemination. Based on this hypothesis, we evaluated whether IIRI may upregulate caspase 3 activity. It is worth noting that IIRI-led epithelial denudation and villus deformation was not only associated with oxidative stress and upregulation of inflammatory cytokines, it was also coupled with enhanced caspase 3 activity. It is likely that caspase 3, an executioner of apoptosis (Akhigbe and Ajayi, 2020), was activated by IIRI-induced oxido-inflammatory response, resulting in the cleavage of downstream death substrates and amplification of upstream death cascade that culminate in apoptosis (Akhigbe and Ajayi, 2020). To this end, it is safe to conclude that activation of caspase 3 is essential in the pathogenesis of epithelial barrier damage, enteric bacterial translocation, and hepatic injury in IIRI. Interestingly, MMOLE promoted epithelial restitution and militated against epithelial barrier damage, enteric bacterial translocation, and hepatic injury in IIRI. Thus, the protective activity of MMOLE in IIRI is via downregulation of oxidative stress-dependent caspase 3 activation.

The observed biological activities of MMOLE may be ascribed to its constituents bioactive molecules, especially hydrazine, 9-octadecenoic acid, 1,3-dioxolane, oleic acid, and nonadecanoic acid. Hydrazine has been reported to exert antioxidant antimicrobial activities (Shen et al., 2010). Also, 9-octadecenoic acid has been demonstrated to exert antimicrobial activities against gram positive and gram negative bacteria (Stenz et al., 2008; Garba and Garba, 2017). In addition, 1,3-dioxolane and its derivatives have been reported to possess radical scavenging and antimicrobial activities (Nobre et al., 2014). This molecule and its derivatives have also been demonstrated to act as effective modulators to combat multidrug resistance (Schmidt et al., 2007), thus improving their antimicrobial activities. Oleic acid has been shown to exert anti-inflammatory (Earlia et al., 2019) and antibacterial activities (McGaw et al., 2002; Zheng et al., 2005). The antibacterial property of thiosemicarbazone (Khan et al., 2014) may also contribute to the biological activities of MMOLE.

## 5 Conclusion

In conclusion, methanolic *M. oleifera* leaf extract protects against epithelial mucosal barrier disruption, bacterial translocation, and hepatic injury caused by IIRI via downregulation of oxidative stress-mediated caspase 3 activation and the antimicrobial activities of its constituents bioactive molecules. This study has some limitations. First, GC-MS, instead of high performance liquid chromatography (HPLC), was used to characterize the constituent bioactive molecules of methanolic *M. oleifera* leaf extract due to availability. This possibly provided a limited view of the active compounds in the extract. Also, the pathways evaluated are limited. Future studies evaluating the roles of other apoptotic pathways in IIRI, the bioactive components of methanolic *M. oleifera* leaf extract using HPLC, and the protective potentials of methanolic *M. oleifera* leaf extract against identified pathways are recommended.

## Data Availability

The original contributions presented in the study are included in the article/Supplementary Material, further inquiries can be directed to the corresponding author.

## Abbreviations

AST, aspartate transaminase; ALT, alanine transferase; Caspase 3, cysteine-aspartic protease 3; EMB, eosin-methylene blue; GC-MS, Gas chromatography-mass spectrophotometric; GGT, gamma-glutamyl transferase; GPx, glutathione peroxidase; GSH, reduced glutathione; Hb, haematocrit ount; IL-6, interleukin-6; IR, ischaemia/reperfusion; IIRI, intestinal ischaemia/reperfusion injury; LDH, lactate dehydrogenase; MCCA, Mac Conkey agar; MCHC, mean corpuscular haemoglobin concentration; MCV, mean corpuscular volume; MDA, malondialdehyde; MODS, multiple organ dysfunction syndrome; MPO, myeloperoxidase; NA, nutrient agar; NF-kB, nuclear factor kappa-light-chain-enhancer of activated B cells; PCV, packed cell volume; RBC, red blood cell count; ROS, reactive oxygen species; WBC, white blood cell count; SIRS, systemic inflammatory response syndrome (SIRS); SOD, superoxide dismutase; TNF-α, tumour necrotic factor-α.
