# Supplementary material for: Methanolic Moringa oleifera leaf extract protects against epithelial barrier damage and enteric bacterial translocation in intestinal I/R: Possible role of caspase 3
Source: Front Pharmacol. 2022 Sep 23;13:989023. doi: 10.3389/fphar.2022.989023 (PMC9546449; doi:10.3389/fphar.2022.989023)
Supplement: Supplementary file 1 [file DataSheet1.doc]

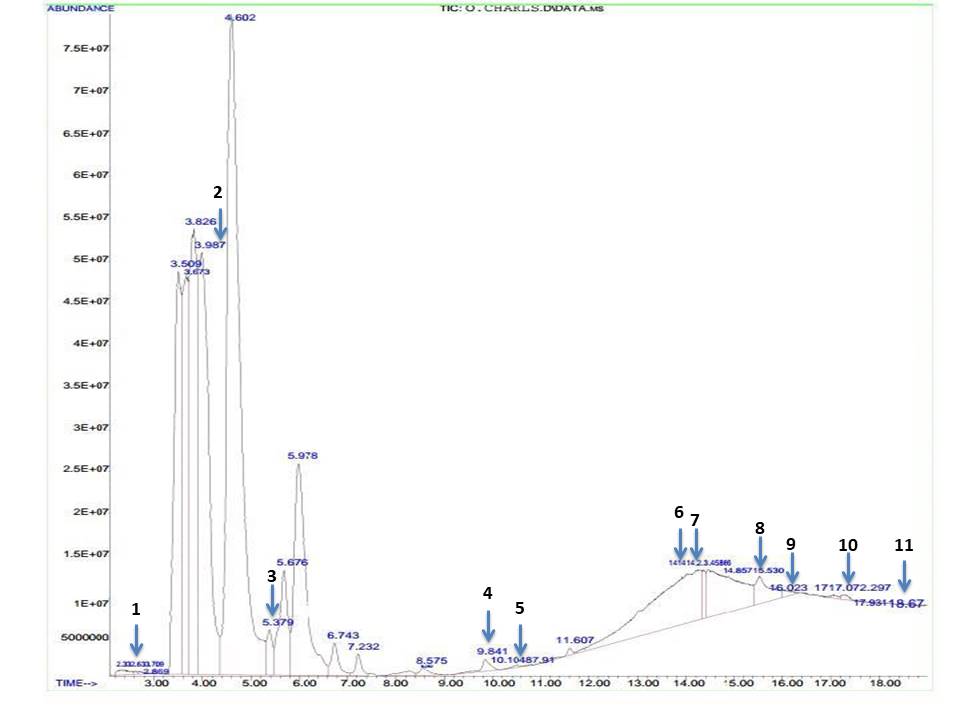


Supplementary Figure 1: GC-MS analysis of methanolic *Moringa oleifera* leaf extract

1: Thiosemicarbazone, 2: hydrazine, 3: 1,3-dioxolone, 4: octanoic acid, 5: 1,3-benzenediamine, 6: 9-octadecenoic acid, 7: oleic acid, 8: nonadecanoic acid, 9: 3-undecanone, 10: phosphonic acid, 11: cyclopentanecarboxylic acid

Phosphonic acid

O

P

R

OH

OH

Thiosemicarbazone

3

S

N

R

N

R

4

R

5

N

R

2

R

1

Hydrazine 1,3- dioxolane

N

N

H

H

H

H

N

N

H

H

H

H


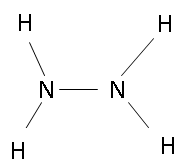


O

O

OH

O

Octanoic acid

O

OH

1,3-benzenediamine Cyclopentanecarboxylic acid

H_2_N

NH_2_

3-undecanone Nonadecanoic acid

OH

O

CH_3_

CH_3_

O

Oleic acid

O

O

9-octadecenoic acid

O

OH

Supplementary Figure 2: Chemical structures of the bioactive molecules of methanolic *Moringa oleifera* leaf extract
