# Supplementary material for: Methanolic Moringa oleifera leaf extract protects against epithelial barrier damage and enteric bacterial translocation in intestinal I/R: Possible role of caspase 3
Source: Front Pharmacol. 2022 Sep 23;13:989023. doi: 10.3389/fphar.2022.989023 (PMC9546449; doi:10.3389/fphar.2022.989023)
Supplement: Supplementary file 2 [file Table1.doc]

Supplementary Table 1: Organic constituents of methanolic *Moringa oleifera* leaf extract revealed by GC-MS

| **S/N** | **Retention time** | **Compound** | **Quality** | **Molecular formula** | **Molecular weight (g/mol)** | **Percentage composition**  **(%)** |
| --- | --- | --- | --- | --- | --- | --- |
| **1** | 2.631 | Thiosemicarbazone | 37 | H2NC(S)NHN=CR_2_ | 236.30 | 0.03 |
| **2** | 3.987 | Hydrazine | 4 | H6N2O | 50.06 | 14.50 |
| **3** | 5.379 | 1,3-Dioxolane | 9 | C3H6O2 | 74.08 | 2.92 |
| **4** | 9.841 | Octanoic acid | 45 | C8H16O2 | 144.21 | 0.33 |
| **5** | 10.48 | 1,3-Benzenediamine | 35 | C7H10N | 122.17 | 0.06 |
| **6** | 14.235 | 9-octadecenoic acid | 97 | C18H38O2 | 282.46 | 8.74 |
| **7** | 14.235 | Oleic acid | 96 | C18H34O2 | 282.47 | 2.14 |
| **8** | 15.530 | Nonadecanoic Acid | 86 | C21H42O2 | 326.60 | 1.24 |
| **9** | 16.023 | 3-undecanone | 43 | C11H22O | 170.29 | 0.18 |
| **10** | 17.072 | Phosphonic Acid | 38 | H2O3P+ | 80.98 | 0.09 |
| **11** | 18.676 | Cyclopentanecarboxylic acid | 38 | C6H10O2 | 114.14 | 0.02 |
